# Highly Efficient Reproducible Perovskite Solar Cells Prepared by Low-Temperature Processing

**DOI:** 10.3390/molecules21040542

**Published:** 2016-04-23

**Authors:** Hao Hu, Ka Kan Wong, Tom Kollek, Fabian Hanusch, Sebastian Polarz, Pablo Docampo, Lukas Schmidt-Mende

**Affiliations:** 1 Department of Physics, University of Konstanz, 78457 Konstanz, Germany; hao.hu@uni-konstanz.de (H.H.); ka.kan.wong@uni-konstanz.de (K.K.W.); 2 Department of Chemistry, University of Konstanz, 78457 Konstanz, Germany; tom.kollek@uni-konstanz.de (T.K.); Sebastian.Polarz@uni-konstanz.de (S.P.); 3 Department of Chemistry, University of Munich (LMU), 81377 Munich, Germany; fabian.hanusch@cup.lmu.de (F.H.); pablo.docampo@cup.uni-muenchen.de (P.D.)

**Keywords:** perovskite solar cells, perovskite solar cell structures

## Abstract

In this work, we describe the role of the different layers in perovskite solar cells to achieve reproducible, ~16% efficient perovskite solar cells. We used a planar device architecture with PEDOT:PSS on the bottom, followed by the perovskite layer and an evaporated C_60_ layer before deposition of the top electrode. No high temperature annealing step is needed, which also allows processing on flexible plastic substrates. Only the optimization of all of these layers leads to highly efficient and reproducible results. In this work, we describe the effects of different processing conditions, especially the influence of the C_60_ top layer on the device performance.

## 1. Introduction

Perovskite solar cells have attracted a lot of attention since their first report in 2009 [1]. Within five years, the reported efficiency has exceeded over 20% [2,3], which makes it an extremely promising and fast developing candidate for a next generation solar cell. This exciting progress is attributed to the application and exploration of organic-inorganic lead-halide perovskite. Perovskite is a large class of materials, named after the Russian mineralogist Lev Perovski. This special lead-halide perovskite possesses almost all desired properties for light-harvesting solar cell materials, such as suitable direct band-gap [4,5,6,7], strong light absorption ability [8,9], long charge carrier diffusion length [10,11,12,13] and so on. It is exceptional that it forms an excellent light harvesting semiconductor material from solution processing.

Perovskite solar cells were first fabricated using dye-sensitized solar cell architecture with the perovskite as a thin absorber layer on top of a mesoporous TiO_2_ electron acceptor layer [14,15,16]. Soon, it became clear that the mesoporous TiO_2_ is not needed for charge separation as exciton binding energy in perovskites is very low (comparable to inorganic semiconductors) and charge diffusion lengths for electrons and hole are large and relatively balanced [12,13]. Consecutively, planar n-i-p perovskite solar cell architectures have successfully been fabricated [17,18,19]. However, in the two types of architecture above, the high temperature sintering process of the TiO_2_ film (mesoporous or planar) and serious hysteresis behavior [20,21,22,23] have become an obstacle to potential commercial application.

In contrast, an inverted perovskite p-i-n planar heterojunction can be fabricated by a low-temperature process when using poly-(2,3-dihydrothieno-1,4-dioxin)–poly(styrene -sulfonate) (PEDOT:PSS) and C_60_ as hole and electron extraction layers. Such cells are compatible with commercial roll-to-roll fabrication techniques also on flexible substrates, and the hysteresis phenomenon is also greatly suppressed in this cell layout [24,25,26,27,28]. Therefore, a lot of efforts have been focused on achieving the full potential of p-i-n type perovskite solar cells. Several film deposition methods have been used to fabricate perovskite films in flat p-i-n heterojunction solar cells such as two-step dipping [29] or casting [27,30], and a one-step solution processing method [25,31,32,33]. In the latter case, different solvent engineering treatments [34], pre or post annealing treatments [35] or additives (such as *i.e.*, H_2_O [36], [6,6]-phenyl-C61-butyric acid methyl ester (PCBM) [37], hydriodic acid [26], 1,8-diiodooctane (DIO) [38] and so on) have been studied to achieve a uniform and pin-hole free perovskite film. However, despite the great progress, it still remains a difficult task to deposit compact high-quality perovskite films.

In this article, a novel vacuum assisted one-step solution (VAOS) method is reported for producing high-quality perovskite layers. After optimization, over 16% efficiency is achieved with a fill factor around 80%. This method is highly reproducible with over 15% statistical average and over 16% maximum efficiency.

## 2. Results

**Figure 1:**
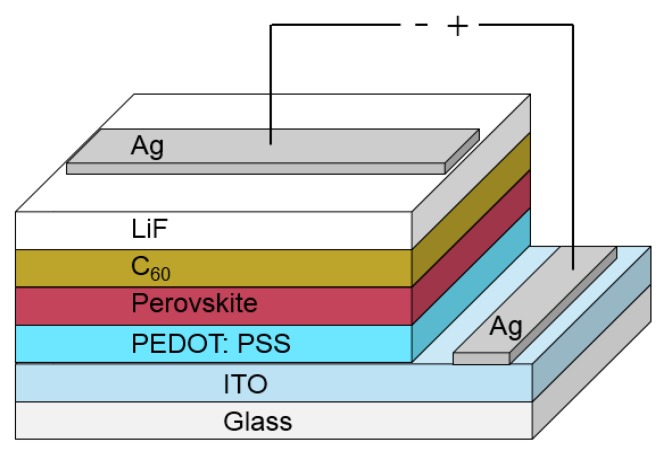
In this figure, a schematic of the used device structure containing all layers is presented. The layer thicknesses are not in scale. The different layer thicknesses are presented in Section 4.2 for optimized solar cell performance.

A schematic of the device structure used for optimizing the solar cell performance is shown in Figure 1. We have prepared our devices on glass substrates with a commercial indium-tin-oxide (ITO) layer. As the first layer, we spin-coat PEDOT:PSS, which is used as a base for the perovskite layer that is spin-coated afterwards. The following layers, such as C_60_, LiF and Ag top contact are consecutively evaporated. Our measurements show that the preparation of the perovskite layer and the evaporation of the C_60_ layer play significant roles for the final device performance. Therefore, we will focus here in more detail on these layers. All the other layers are processed in a common way and details are described in Section 4.

### 2.1. Perovskite Layer

The preparation of perovskite film plays a key role in the fabrication of the whole device. It has been widely accepted that the perovskite crystalization process needs to be carefully tuned to get compact films [39,40]. Perovskite has strong polarity and is prone to forming uncontinuous films. Different precursor solutions and additives have been reported to achieve better film morphology by speeding up [41] or slowing down [38,42,43] the crystallization dynamics. Here, we fabricate a compact and pin-hole free perovskite layer by accelerating the crystallization process by the vacuum assisted one-step solution method (VAOS).

**Figure 2:**
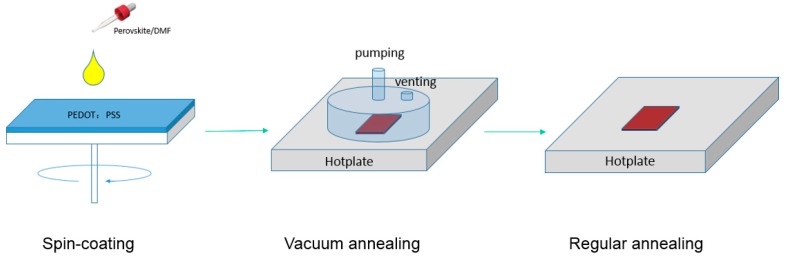
Schematic of the vacuum assisted one-step solution (VAOS) preparation method of the perovskite film.

**Figure 3:**
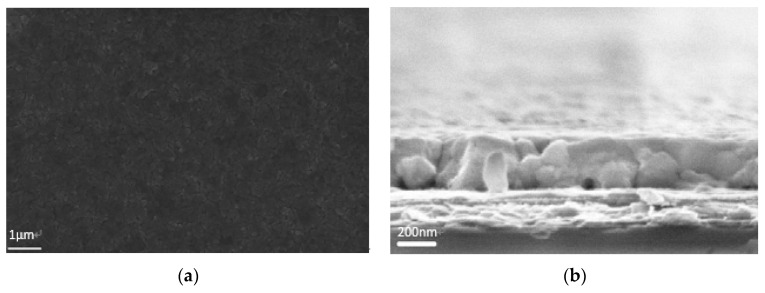
Smooth, uniform, pin-hole free perovskite film (**a**) top-view; (**b**) cross-section SEM images.

The VAOS method diagram is shown in Figure 2. Simply, mixed halide perovskite solution is spin-coated on ITO/PEDOT:PSS substrates in the glovebox with less than 5 ppm moisture level. Then, the substrates are transferred immediately onto a hotplate in a vacuum chamber. A combination of higher temperature and a vacuum removes very quickly all residual solvent leading to a fast crystallization process in the film, which is expected to be faster than the commonly used temperature annealing without additional vacuum. In Figure 3, the top-view and cross-section SEM image of perovskite films are presented.

The film is quite compact and no pin-holes were observed on the top-view image. On the cross-section image, the perovskite layer on ITO/PEDOT:PSS has a uniform thickness of 300 nm as demonstrated in Figure 3b).

Compared to other methods, because of the fast crystallization process, the perovskite crystals are relatively small and have a wide range of size distribution (from around 100 nm to 1 µm). On the other hand, the crystallization nuclei are forced to pack closely and form a very flat and continuous, virtually pin-hole free film. It is worth mentioning that the film is so flat that no obvious undulation could be observed even for an only 70 nm thick perovskite thin film (see Supplementary Material Figure S1), which enables simple formation of semi-transparent perovskite solar cells by reducing the film thickness. During vacuum annealing, the perovskite film color changed from colorless to red-brown, and longer vacuum annealing leads to a notably darker color. It has been found the vacuum annealing needs to be carefully optimized regarding annealing time and temperature, as soon as fully conversion is completed, further annealing could be harmful leading to perovskite decomposition [44,45]. In our experiments, a vacuum annealing directly after spin-coating of the perovskite film for 4 min at 80 °C leads to the most efficient device performance.

### 2.2. C_60_ Layer

#### 2.2.1. Influence of C_60_ Layer Thickness

**Figure 4:**
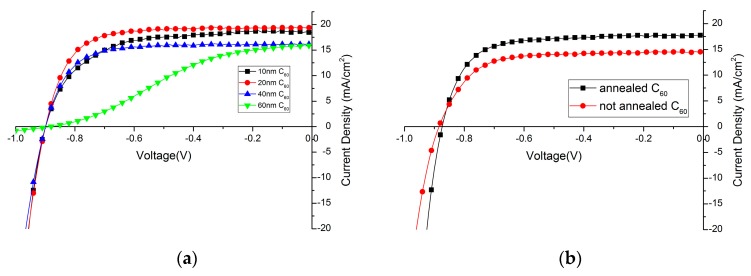
Influence of C_60_ layer (**a**) thickness; and (**b**) annealing effect on device performance.

C_60_ layer is used to act as a selective electron extraction layer in the solar cell. Its thickness needs to be carefully controlled, as it needs to be thick enough to form a compact layer over the perovskite film, but a too thick C_60_ layer will increase the series resistance of the device. C_60_ layers with different thickness have been thermally evaporated on top of the perovskite film. In Figure 4a, the influence of the C_60_ layer thickness on the device performance is shown. Detailed solar cell parameters are summarized in Table 1. Clearly, 20 nm C_60_ is already enough as a hole-blocking layer, and doubling its thickness greatly impedes its ability to transfer electrons which can be observed in a low fill factor and also lower photocurrent density. If the layer is too thin, the fill factor and photocurrent density also decreases slightly, which is assumed to be due to a not full coverage of the perovskite film without any pinholes. Compared to PCBM, which is usually around 100 nm [30], C_60_ is more electron-conductive [46] and requires a very flat perovskite film as a layer underneath. Only, in this case, it is possible to have thin, but compact and pinhole-free films.

#### 2.2.2. Influence of C_60_ Annealing

It is found that the performance of solar cells will benefit from a short annealing treatment after deposition of C_60_ layer (10 min at 90 °C in N_2_ atmosphere), mainly due to the increase of photocurrent. (Figure 4b, Table 2). It has been reported that PCBM strongly diffuses through films when annealed. It is expected that also the C_60_ diffuses with annealing and changes the perovskite/C_60_ interface promoting charge transfer [30]. Additional aggregation might increase the charge carrier mobility of the C_60_ film, thus improving charge collection at the electrode.

### 2.3. Further Optimization

**Figure 5:**
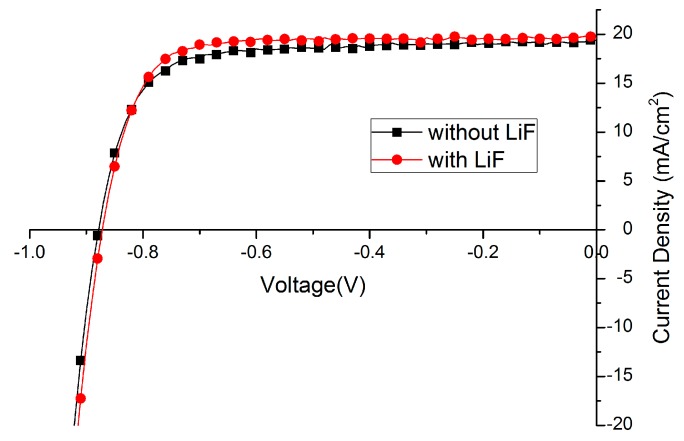
Effect of lithium fluoride (LiF) layer on Current density -Voltage (J–V) curve.

Lithium fluoride (LiF), bathocuproine (BCP) or Ca are widely used as the interfacial layer between electron transport layer (ETL) and metal back contact. In this paper, the effect of LiF is studied. Amazingly, the fill factor (FF) of solar cells could be improved from 70%–75% to around 80% just by depositing 1 nm LiF before Ag evaporation (Figure 5, Table 3). Such an effect has been reported [34,47,48], implying the energy mismatch between cathode and electron transporting layer is compensated by the formation of dipoles with LiF insertion layer. We also expect that the LiF layer protects the layers underneath when the silver is evaporated on top. Less silver will diffuse into the film forming high conducting channels leading to a recombination reducing fill-factor.

### 2.4. Optimized Solar Cell Performance and Statistical Results

**Figure 6:**
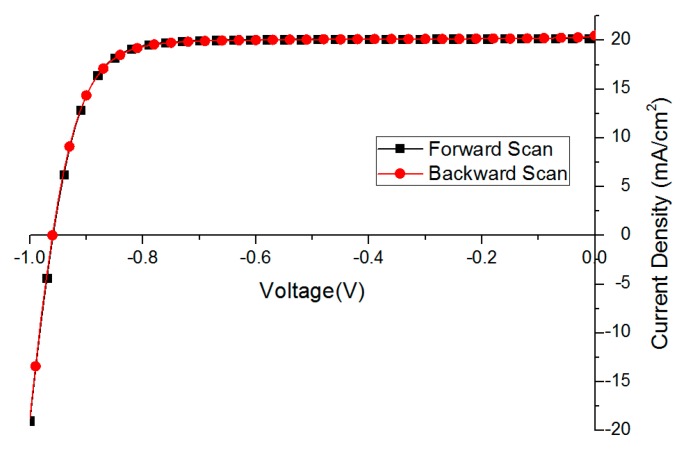
The best solar cell performance without hysteresis behavior (scan rate 0.1 V/s).

Based on the optimization results mentioned above, we deposited 30 perovskite films and fabricated solar cells in successive three batches. The performance of the best fully optimized solar cell is presented in Figure 6 and Table 4 with no obvious hysteresis behavior.

The FF of the backward scan is lower (79.4 *vs.* 81.0 in Table 4) mainly due to a steeper current drop at 0 bias points, which is attributed to certain charge accumulation.

**Figure 7:**
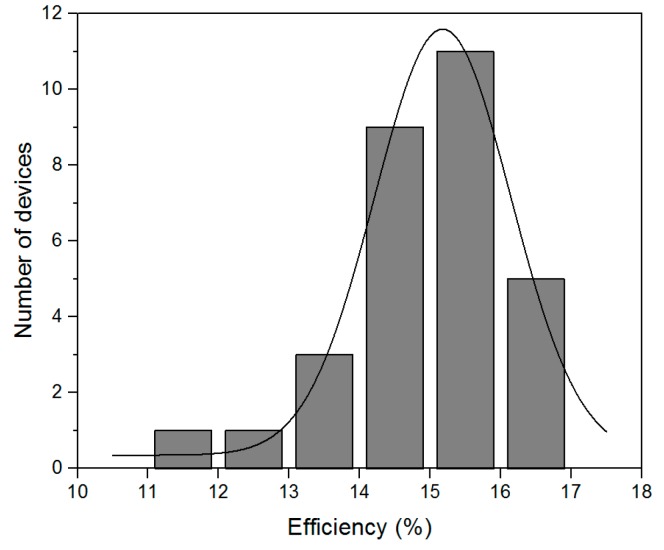
The statistical distribution of 30 solar cell efficiencies fabricated in three successive processes.

The statistical distribution of these results is shown in Figure 7. As can be seen, our fabrication method is quite reproducible, and over 80% solar cells have over 14% efficiency. The inefficient results are considered to come from intrinsic flaw-prone spin-coating process which, with the oversaturated precursor solution, sometimes induces macroscopic pinholes.

## 3. Discussion

The optimization results in the previous sections clearly indicate that not only does the perovskite layer preparation play a significant role in the final device performance, but also the interfacial layers. With the described method, we could achieve a champion device efficiency over 16%. Most cells showed a performance exceeding 14% efficiency.

It has been considered that large perovskite crystals are superior to small ones in solar cells, as they have less grain boundaries, thus less recombination and longer charge carrier life time. However, in our case, we show that, even with small perovskite crystals, solar cells still work very efficiently with an over 80% fill factor with optimized C_60_ and LiF layer. The presented novel VAOS deposition method could produce ultra-thin, ultra-flat and compact perovskite film, and we believe it will contribute to this flourishing research area.

## 4. Materials and Methods

### 4.1. Preparation of the Precursor

Methylammonium iodide (MAI) was synthesized as reported [49]. In short, 24 mL of methylamine solution (33% in ethanol, Sigma-Aldrich, Schnelldorf, Germany) was diluted with 100 mL of absolute ethanol in a 250 mL round-bottom flask. In addition, 10 mL of hydroiodic acid (33 wt%) was added under constant stirring into the flask. After reacting one hour at room temperature, the solvents were removed by rotary evaporation. The obtained white solid was washed with dry diethyl ether and finally recrystallized with ethanol. To prepare mixed-halide perovskite precursor solutions, PbI_2_, PbCl_2_ and MAI were mixed in anhydrous dimethylformamide (DMF) (99.9%) with a molar ration of 1:1:4 [50]. The solution concentration is around 40 wt%. The solutions were stirred at room temperature overnight in a glovebox and stored there for use.

### 4.2. Fabrication of the Devices

The ITO-coated glass substrates (15 Ω per sq) were ultrasonic cleaned for 5 min by detergent, acetone and isopropanol successively. Then, they were treated with oxygen plasma for 7 min. After that, around 30 nm PEDOT:PSS (Clevios™ VP AI 4083) film was spin-coated on top (Supplementary Material Figure S2) and then annealed at 180 °C for 5 min. Then, the substrates were transferred to a glovebox with moisture and oxygen level both lower than 5 ppm. The prepared precursor solution was spin-coated on the substrates with 3000 rpm for 20 s. Then, the films were transferred immediately to a vacuum chamber on a hotplate at 90 °C. After evacuating, the films turned red-brown and then were further annealed at normal pressure at 100 °C for full conversion to perovskite. Finally, a C_60_ layer, LiF layer and Ag back contact were thermally evaporated consecutively on top of the spin-coated perovskite film. Layer thickness details are summarized in Table 5.

### 4.3. Device Characterization

The morphology of the perovskite film were characterized using a scanning electron microscopy (SEM) (Zeiss Neon 40 EsB, Oberkochen, Germany) operated at 5 kV accelerating voltage. Current density-voltage (J–V) measurements were performed using a Keithley 2400 SourceMeter (Tektronix GmbH, Keithley Instruments, Germering, Germany) controlled through a self-written LabView program (National Instruments, Austin, Texas, US). Cells were illuminated via a LOT-Oriel LS0106 solar simulator (LOT, Darmstadt, Germany) through a shadow mask with a resulting active area of 0.09 cm^2^. Light intensities were calibrated with a certified Si reference solar cell (Fraunhofer Institute, Freiburg, Germany) with a KG5 filter (Schott, Mainz, Germany). The light intensity of the solar simulator was around 0.95 sun.

## Figures and Tables

**Table 1:** Effect of C_60_ layer thickness on device performance.

C_60_ Layer Thickness	PCE (%)	FF	V_OC_ (mV)	J_SC_ (mA/cm^2^)
10 nm	10.5	63.6	899	18.4
20 nm	12.2	72.2	899	19.4
40 nm	9.3	65.9	902	16.1
60 nm	4.8	35.0	884	15.8

PCE: power conversion efficiency, FF: fill factor, V_OC_: open circuit voltage, J_SC_: short circuit current density.

**Table 2:** Annealing effect of C_60_ layer.

Treatment	PCE (%)	FF	V_OC_ (mV)	J_SC_ (mA/cm^2^)
Annealed C_60_	11.5	71.0	874	17.7
Non-annealed C_60_	9.35	69.5	885	14.5

PCE: power conversion efficiency, FF: fill factor, V_OC_: open circuit voltage, J_SC_: short circuit current density.

**Table 3:** Effect of lithium fluoride (LiF) layer on solar cell performance.

Device	PCE (%)	FF	V_OC_ (mV)	J_SC_ (mA/cm^2^)
Without LiF	12.6	74.4	878	19.4
With LiF	13.3	78.7	872	19.6

PCE: power conversion efficiency, FF: fill factor, V_OC_: open circuit voltage, J_SC_: short circuit current density.

**Table 4:** The best perovskite solar cell performance after full optimization.

Scan direction	PCE (%)	FF	V_OC_ (mV)	J_SC_ (mA/cm^2^)
Backward scan	16.4	79.4	960	20.5
Forward scan	16.4	81.0	959	20.2

PCE: power conversion efficiency, FF: fill factor, V_OC_: open circuit voltage, J_SC_: short circuit current density.

**Table 5:** Layer deposition technique and thickness of different layers in the optimized solar cell geometry.

Layer	Deposition Method	Thickness
Glass	-	1.1 mm
ITO	-	around 150 nm
PEDOT:PSS	Spin-coated	30 nm
Perovskite	Spin-coated	300 nm
C_60_	Evaporated	20 nm
LiF	Evaporated	1 nm
Ag	evaporated	100 nm

## Supplementary Materials

The following are available online at: http://www.mdpi.com/1420-3049/21/4/542/s1, Figure S1: Ultraflat perovskite film with around 70 nm thickness deposited with the VAOS method, Figure S2: Cross-section SEM graph of PEDOT:PSS film.
