# Supplementary material for: Highly Efficient Reproducible Perovskite Solar Cells Prepared by Low-Temperature Processing
Source: Molecules. 2016 Apr 23;21(4):542. doi: 10.3390/molecules21040542 (PMC6274524; doi:10.3390/molecules21040542)
Supplement: Supplementary file 1 [file molecules-21-00542-s001.pdf]

Supplementary Material: Highly Efficient Reproducible Perovskite Solar Cells Prepared by Low-Temperature Processing

Hao Hu, Ka Kan Wong, Tom Kollek, Fabian Hanusch, Sebastian Polarz, Pablo Docampo and Lukas Schmidt-Mende

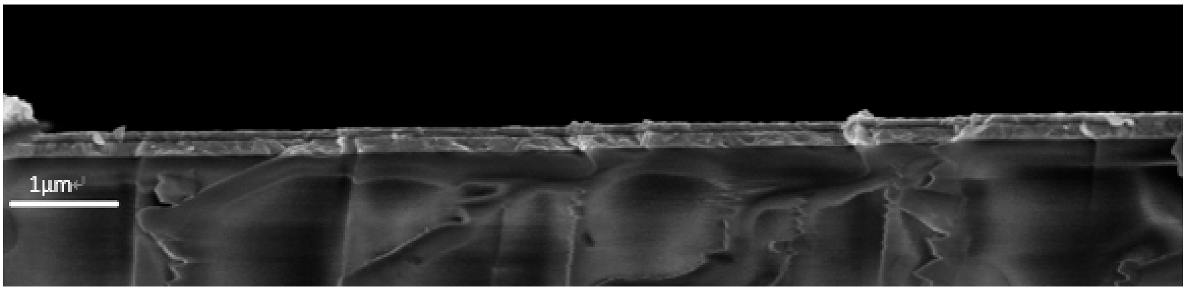

Figure S1. Ultraflat perovskite film with around 70 nm thickness deposited with VAOS method.

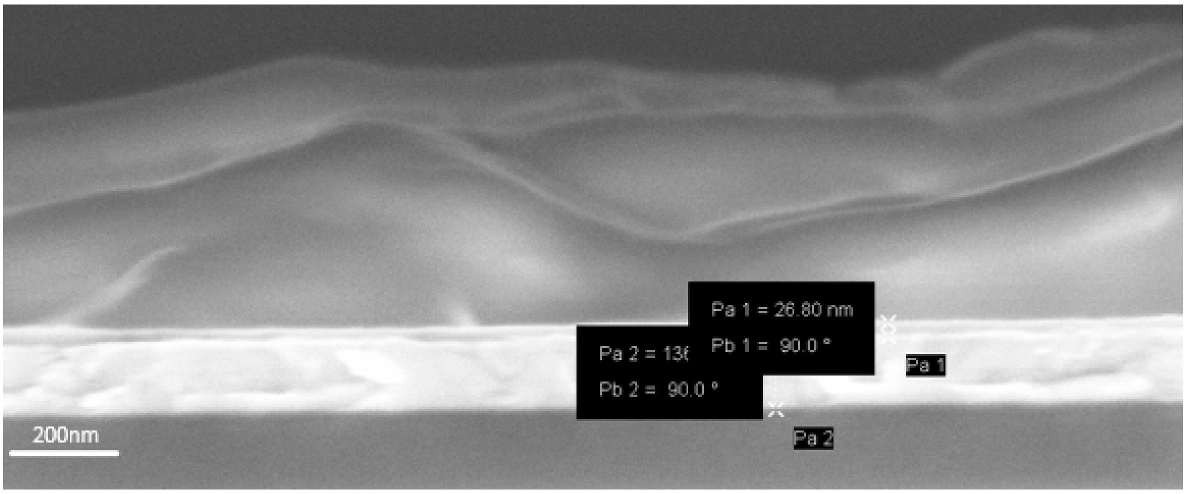

Figure S2. Cross-section SEM graph of PEDOT:PSS film (PEG deposited on top for contrast).
